# Exploration of exosomal microRNA expression profiles in pigeon ‘Milk’ during the lactation period

**DOI:** 10.1186/s12864-018-5201-0

**Published:** 2018-11-20

**Authors:** Yao Ma, Siyuan Feng, Xun Wang, Izhar Hyder Qazi, Keren Long, Yi Luo, Guojun Li, Chunyou Ning, Yixin Wang, Silu Hu, Juan Xiao, Xiaokai Li, Dan Lan, Yaodong Hu, Qianzi Tang, Jideng Ma, Long Jin, Anan Jiang, Mingzhou Li

**Affiliations:** 1 Institute of Animal Genetics and Breeding, College of Animal Science and Technology, Sichuan Agricultural University, Chengdu, 611130 Sichuan China; 2 Department of Veterinary Anatomy and Histology, Faculty of Bio-Sciences, Shaheed Benazir Bhutto University of Veterinary & Animal Sciences, Sakrand, Sindh 67210 Pakistan

**Keywords:** Pigeon ‘milk’, Exosomes, miRNAs, Small RNA-seq

## Abstract

**Background:**

Pigeon crop has the unique ability to produce a nutrient rich substance termed pigeon ‘milk’ (PM), which has functional resemblance with the mammalian milk. Previous researches have demonstrated that a large number of exosomes and exosomal miRNAs exist in mammalian milk, and many of them are associated with immunity, growth and development. However, to date, little is known about the exosomes and exosomal miRNAs in PM.

**Results:**

In this study, we isolated the exosomes from PM and used small RNA sequencing to investigate the distribution and expression profiles of exosomal miRNAs. A total of 301 mature miRNAs including 248 conserved and 53 novel miRNAs were identified in five lactation stages i.e. 1d, 5d, 10d, 15d, and 20d. From these, four top 10 conserved miRNAs (cli-miR-21-5p, cli-miR-148a-3p, cli-miR-10a-5p and cli-miR-26a-5p) were co-expressed in all five stages. We speculate that these miRNAs may have important role in the biosynthesis and metabolism of PM. Moreover, similar to the mammalian milk, a significant proportion of immune and growth-related miRNAs were also present and enriched in PM exosomes. Furthermore, we also identified 41 orthologous miRNAs group (giving rise to 81 mature miRNA) commonly shared with PM, human, bovine and porcine breast milk. Additionally, functional enrichment analysis revealed the role of exosomal miRNAs in organ development and in growth-related pathways including the MAPK, Wnt and insulin pathways.

**Conclusions:**

To sum-up, this comprehensive analysis will contribute to a better understanding of the underlying functions and regulatory mechanisms of PM in squabs.

**Electronic supplementary material:**

The online version of this article (10.1186/s12864-018-5201-0) contains supplementary material, which is available to authorized users.

## Background

Unlike the most avian species, the pigeons (*Columba livia*) are among few representative birds which have the ability to produce a unique nutritive substance called pigeon ‘milk’ (PM) for the sustenance and growth of their squabs. PM is a curd like cheesy nutritive solution and has many functional similarities with the mammalian milk in terms of delivery of nutritional and immunogenic benefits and subsequent growth and development of young ones [1]. Despite such similarities, one contrasting fascination is that PM is produced by both female and male pigeons; however, there is dearth of scientific literature addressing the underlying mechanisms of these processes. The production of PM is highly specialized process, it is well established that, alike mammals, the prolactin (lactogenic hormone) regulates the production of PM in pigeons [2]. However, histological studies on lactating pigeon crop tissue have showed that the process is structurally orthogonal to conventional mammalian lactation since the pigeon crop is non-glandular and secretory processes seem to be absent in it [3–5]. Gillespie et al. in their genome-wide pigeon crop transcriptome study have investigated the underlying molecular mechanism of pigeon milk production [6]. Their findings have revealed that the differential expression of cornification related proteins and de novo lipid synthesis genes in pigeon crop during lactation contribute to the highly specialized process that leads to the production of PM. PM consists of lipid enriched keratinocytes (differentiated cornified cells) which after undergoing rapid proliferation have separated from crop sac germinal epithelium [7]. A large number of studies have indicated that the PM contains highly nutritious substances such as proteins, lipids, minerals, carbohydrates and growth factors with diverse biological activities [8–10]. Shetty et al. have reported a stupendously rapid growth in squabs during the lactation period, which provides manifestation for the physiological and evolutionary immensity of PM [11]. In previous functional studies, PM has reportedly stimulated the growth of ovarian cells in hamster [11], caused precocial incisor eruption and eyelid opening in newborn mice [12], and promoted the growth in chickens [13]. Gillespie and colleagues have observed the expression of immune-related gene pathways and significantly increased interferon-stimulated genes in the gut associated lymphoid tissue (GALT) of PM fed chickens [1]. However, one previously conducted study reports that the squabs failed to thrive when they were fed with the nutritional replacement of PM [14]. These results suggest that there are other essential factors other than the nutritional substances in PM that influence the development of young.

MicroRNAs (miRNAs) are evolutionary conserved small non-coding RNAs (~ 22 nt), which are widely involved in the complex cellular mechanisms such as differentiation, proliferation, metabolism and immune development through the post-transcriptional gene regulatory mechanisms [15–17]. In recent years, a large number of miRNAs were found to be present in various biological fluids i.e. breast milk, serum, saliva, and urine [18–20], and these extracellular miRNAs are principally delivered by exosomes. Exosomes are nano-sized membrane vesicles of endocytic origin and have emerged as important mediators of the intercellular communication in immune system and elsewhere by transferring miRNAs [21, 22]. In our previous studies, we have reported that the immune-related exosomal miRNAs were enriched in human [22], porcine [23] and giant panda breast milk [24]. Based on findings of these studies, we speculated and suggested that the exosomal miRNAs may play a significant role in growth and development of infants. However, to date, no study has reported the presence of miRNAs in PM, therefore, the potential physiological/functional role of exosomal miRNAs in PM remains unidentified.

Here, we conjectured that the exosomal miRNAs are enriched in PM and are involved in modulation of physiology and immunity related mechanisms in developing squabs. We isolated the exosomes from PM and examined the expression profiling of exosomal miRNAs using small RNA-seq approach. Furthermore, in order to elucidate the potential role of exosomal miRNAs in PM, we also performed a comparative transcriptomic analysis of exosomal miRNAs in PM and in breast milk of other three representative mammals i.e. human, cattle and pig.

## Results

### Identification of small RNAs-loaded exosomes in PM

**Fig. 1:**
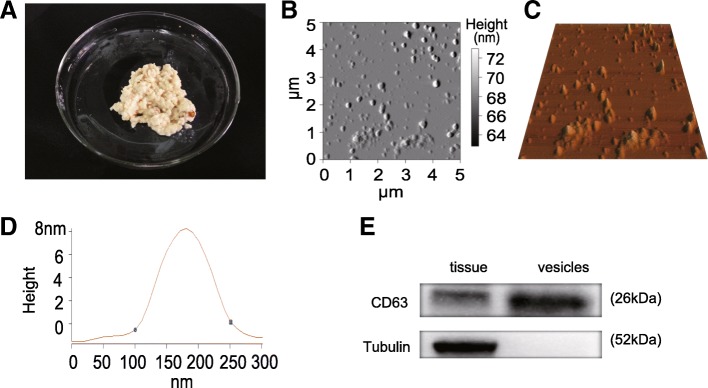
Identification of exosomes in PM. **a** The morphology of PM. **b** 2D AFM image of isolated exosome vesicles in PM. **c** 3D AFM image of isolated exosome vesicles in PM. **d** The line profile of AFM image for a PM exosome vesicle. X- and Y-axes are the width and height of PM exosome vesicle, respectively. **e** The expression of CD63 and Tubulin in the crop tissue and exosome vesicles was determined by Western blotting. It can be seen that the isolated exosome vesicles are positive for CD63 but negative for Tubulin

Exosomes were isolated from PM (Fig. 1a) by ultracentrifugation and were investigated under the atomic force microscope (AFM) at the nanometer scale. The PM contained a substantial amount of exosome-like vesicles (Fig. 1b, c) with an approximate width of 150 nm and a height of 8 nm (Fig. 1d). They exhibited the similar ultrastructural morphology as that of exosomes previously identified in porcine [23] and bovine breast milk [25]. Additionally, as per recommendation of the International Society for Extracellular Vesicles (ISEV) the exosomes were further confirmed by Western blot analysis. As an exosomal marker, the tetraspanin molecule CD63 was detected, while the Tubulin (a cytoskeletal protein) was absent in ultracentrifugation pellets (Fig. 1e). We further identified that these vesicles contained a substantive quantity of small RNAs shorter than 100 nt in length, but no 18S and 28S ribosomal RNA were observed (Additional file 1: Figure S1), which confirmed the presence of small RNAs in exosomes.

### Identification of exosomal miRNAs in PM

To identify the exosomal miRNAs in PM across the lactation periods, we generated a total of 95,476,045 raw reads of seven small RNA libraries (1d-1, 1d-2, 1d-3, 5d, 10d, 15d, 20d). After handling with a series of stringent filters (such as removing low-quality reads, repeated sequences, and adaptor sequences), we obtained a set of 81,219,606 (85%) high-quality reads for miRNA identification. With strict mapping criteria to the pigeon reference genome, retained reads were considered as reliable miRNA candidates (Additional file 2: Table S1). The length distribution peaked at 22~ 24 nt, which is consistent with the animal miRNAs (Additional file 3: Figure S2A).

A total of 230 pre-miRNAs giving rise to 301 mature miRNAs were identified in seven libraries after mapping to the pigeon reference genome (*Columba livia,* ColLiv2) (Table 1). Since, the pigeon miRNA sequences are currently unavailable in miRbase 21.0, the miRNAs were categorized into two groups as per following alignment criteria: 248 miRNAs were mappable to other two known avian species (*Gallus gallus* and *Taeniopygia guttata*) and mammalian miRNAs and are regarded as ‘conserved miRNA’ and prefixed with ‘conserve-cli’. Whereas, 53 miRNAs were unable to be annotated by known avian and mammalian mature miRNAs and satisfied the folding minimum free energy cutoff of RNA hairpin structure and therefore, are called ‘novel miRNAs’ and prefixed with ‘novel-cli’ (Additional file 4: Table S2). Not surprisingly, the read counts of conserved miRNAs were more abundant compared to those of novel miRNAs, which seems to be a common phenomenon across species [26] (Additional file 3: Figure S2B, C).

**Table 1:** Conserved and novel miRNAs detected in exosomes of PM

Type	Pre-miRNA	miRNA-5p	miRNA-3p	Both	Mature miRNA
Conserved miRNA	184	60	60	64	248
Novel miRNA	46	19	20	7	53
Total	230	79	80	71	301

### Immune and growth-related microRNAs are abundant in exosomes of PM

**Fig. 2:**
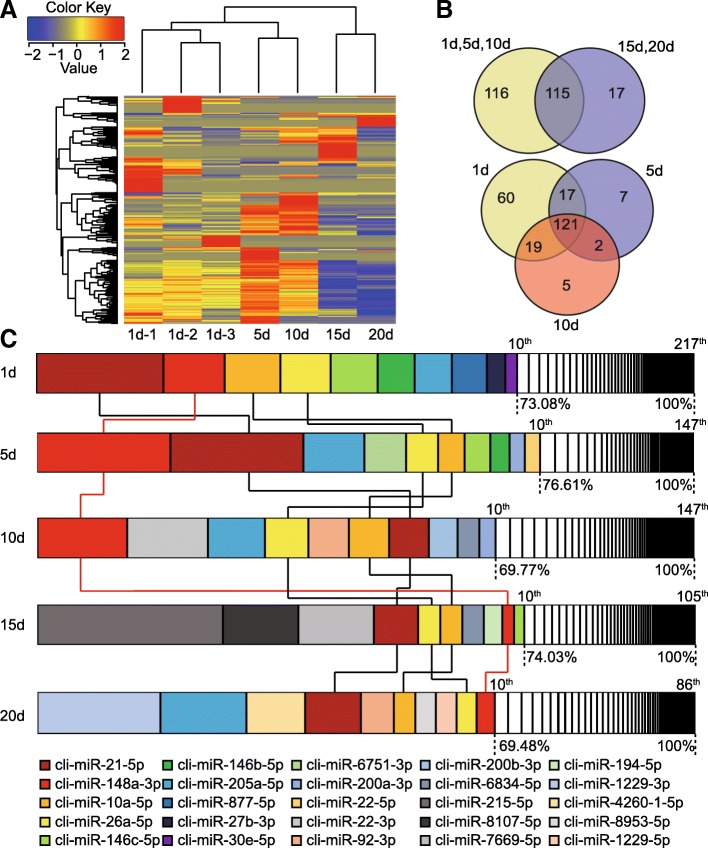
Exosomal miRNAs expression profiles during lactation period. **a** Hierarchical clustering analysis for the normalized expression of conserved miRNAs among seven exosomal miRNA libraries based on Euclidean distance. **b** The number of expressed miRNAs at the two major stages and three early stages. **c** Top 10 miRNAs with the highest expression levels in exosomal miRNA libraries. Box Plot with different color represents the percentage of each miRNA expression in each library. Four co-expressed miRNAs in all 5 lactation stages are connected by lines (cli-miR-148a-3p, a potential biomarker for the quality control of mammalian milk can be seen marked with red line)

To better elucidate the miRNA expression in PM across the lactation periods, we conducted the hierarchical clustering analysis on the annotated conserved miRNA groups. As shown in Fig. 2a, the libraries clustered together based on the lactation time. Two major branches were defined: one representing 1, 5 and 10 days, and other representing 15 and 20 days. This different clustering pattern may correspond to different kinds of miRNAs as well as the different expression patterns of the same miRNAs. Moreover, three biological replicates in 1 day were highly clustered together, which suggested the experimental reliability and highlighted the low variation in miRNAs profiles in PM across the individuals. The two major branches had 115 miRNAs overlapped, accounting for 49.78 and 87.12% in the first three stages (1d, 5d, 10d) and the last two stages (15d, 20d), respectively. Furthermore, 121 miRNAs were shared in 1d, 5d and 10d, only a few miRNAs were uniquely expressed in 5d and 10d, which indicates the time-dependent decreasing tendency in expressed miRNAs species (Fig. 2b). Additionally, we observed an effective correlation between the small RNA-seq and q-PCR expression data for randomly selected miRNAs across five lactation stages. These results shown a similar expression pattern (average Person’s *r* = 0.71), which suggests that the sequencing data were reliable (Additional file 5: Figure S3).

**Fig. 3:**
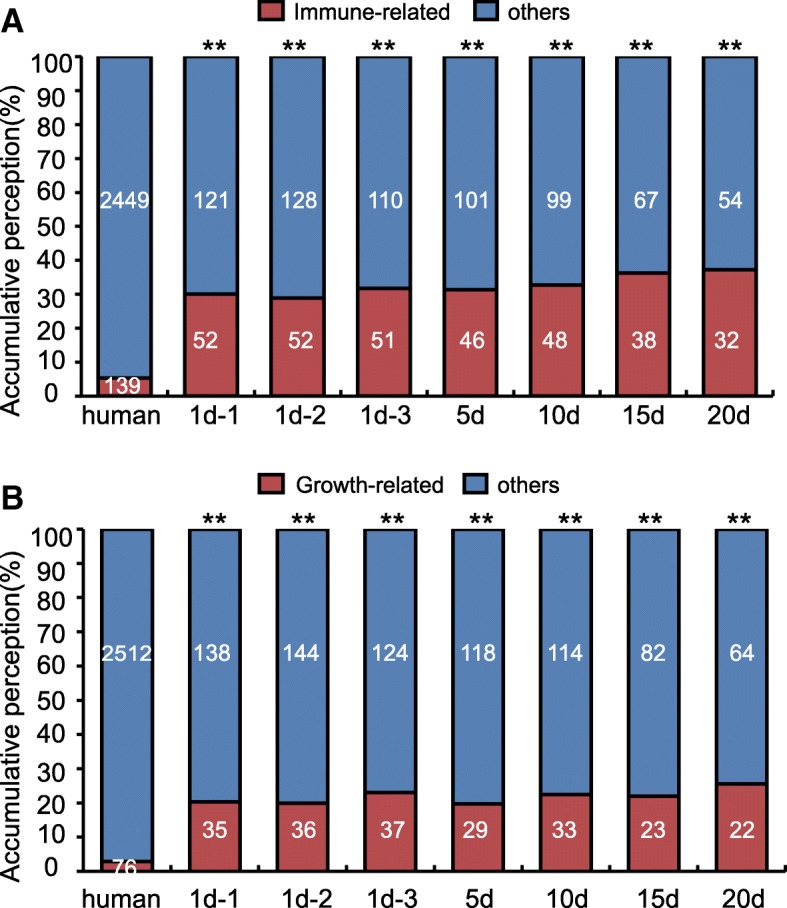
The distribution of immune and growth-related miRNAs in PM. **a** The accumulative perception of immune-related miRNAs in five lactation stages. **b** The accumulative perception of growth-related miRNAs five lactation stages. *χ*^2^-test: Number of immune and growth-related miRNAs and others detected in PM exosomes compared with the total entries in miRBase 21.0. *P < 0.05, **P < 0.01 vs human immune and growth-related miRNAs based on annotation in the Pathway Central database (SABiosciences, MD, USA)

As shown in Fig. 2c, abundant miRNAs concentrated on few miRNAs of PM in five lactation stages. The top ten expressed miRNAs over five lactation stages correspond to 25 kinds of miRNAs and account for more than 69.48% (by total counts) of all the conserved miRNAs. Amid them, four conserved miRNAs (cli-miR-21-5p, cli-miR-148a-3p, cli-miR-10a-5p and cli-miR-26a-5p) were shared by all five lactation stages in top ten positions. These observations suggested that these miRNAs could be the important components of PM. Interestingly, based on annotation of the Pathway Central database (SABiosciences, MD, USA), a substantial proportion of miRNAs (including 3 of top ten co-expressed miRNAs) were designated as immune-related miRNAs. Similar to previous reports on mammalian breast milk [22–24], out of 139 immune-related miRNAs that are most relevant to the function of T-cell and B-cell activation, inflammatory response and autoimmunity, 58 (19.27%) are present and enriched in PM exosomal miRNA libraries (*P* < 10^− 37^, *χ*^2^ - test) (Fig. 3a). For instance, miR-146b, a negative regulator of the innate immune response by targeting NF-*κ*B signaling [27]. miR-27b destabilizes peroxisome proliferator-activated receptor *γ* (*PPARγ*) mRNA, associating with chronic inflammatory diseases [28]. miR-200a-3p is associated with Hodgkin lymphoma [29], and potentially in regulating pathogen recognition in miiuy croaker through TLR1 targeting, an essential component of TLRs in bacterial pathogen defense [30]. The miR-26a, in transformed avian lymphocyte lines, regulates the expression of interleukin-2, which is a significant factor in development, differentiation, proliferation, and homeostasis of T cells [31]. Furthermore, we found that 38 (12.62%) growth-related miRNAs (*P* < 10^− 29^, *χ*^2^ - test) within cell differentiation and development pathways were also present in PM (Fig. 3b). These included miR-21-5p, miR-26a-5p, miR-10a-5p. Amid these, miR-21 has been reported to increase adipogenic differentiation of human adipose tissue-derived mesenchymal stem cells (hASCs) through modulation of TGF-*β* signaling pathway [32]. miR-26a regulates tissue and cell growth and differentiation [33, 34]. miR-10a belongs to the miR-10 family, a miRNA largely involved in development by targeting *Hox* transcripts in several species [35].

### Comparative miRNomes between PM and mammalian milk

**Fig. 4:**
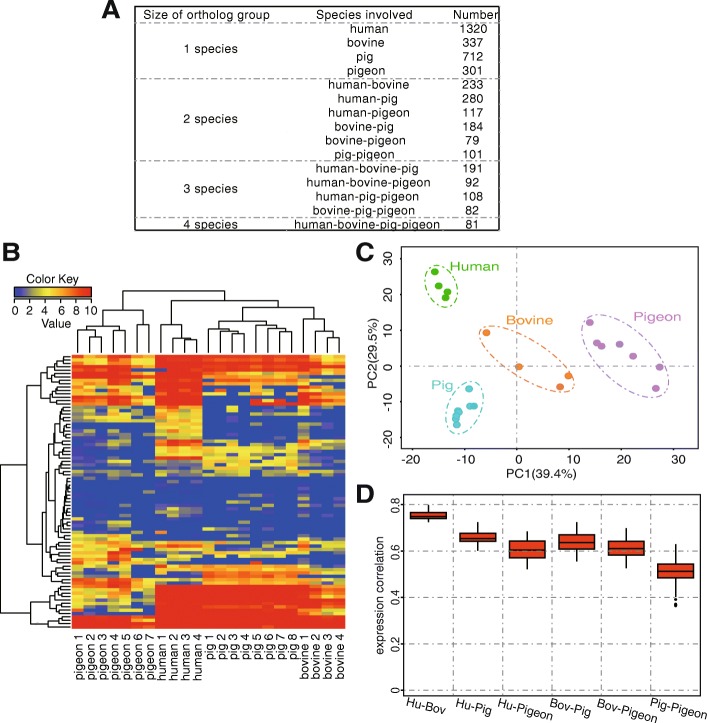
Comparative analysis of conserved miRNAs in PM, human, bovine and porcine breast milks. **a** The ortholog groups of miRNA. **b** Hierarchical clustering analysis of conserved miRNAs across 23 libraries. **c** Principal component analysis (PCA) of conserved miRNAs across all 23 libraries. **d** The pairwise correlation of conserved miRNAs expression among different species

To further enlighten the miRNAs in PM, we identified and compared the conserved orthologous miRNAs in PM and mammalian milk (Additional file 4: Table S2). As depicted in Fig. 4a, out of 301 mature miRNAs in PM, 41 orthologous miRNAs group (giving rise to 81 mature miRNA) were identified across all four species (Additional file 6: Table S3). To examine expression differences among the species, 41 orthologous miRNAs group were further analyzed. The results demonstrated that the libraries of porcine and bovine breast milk were clustered together, and then converged with human breast milk, whilst the libraries of PM constructed a completely separate branch (Fig. 4b). Unsurprisingly, the libraries clustered together based on their respective species of origin. PCA exhibited the specific regions for each species, and the variations in the conserved miRNA expression differed primarily by species (Fig. 4c).

The pairwise correlation of conserved miRNAs expression across the species is shown in Fig. 4d. The expression of conserved miRNAs between human and bovine breast milk exhibited the highest correlation (average Pearson’s *r* = 0.75) compared to each of other libraries, followed by human vs. porcine breast milk, and bovine vs. porcine breast milk. However, the expression correlation of conserved miRNAs between human, bovine, porcine breast milk and PM were 0.60, 0.62 and 0.50, respectively.

### The distinct miRNA expression patterns of PM during different lactation stages

**Fig. 5:**
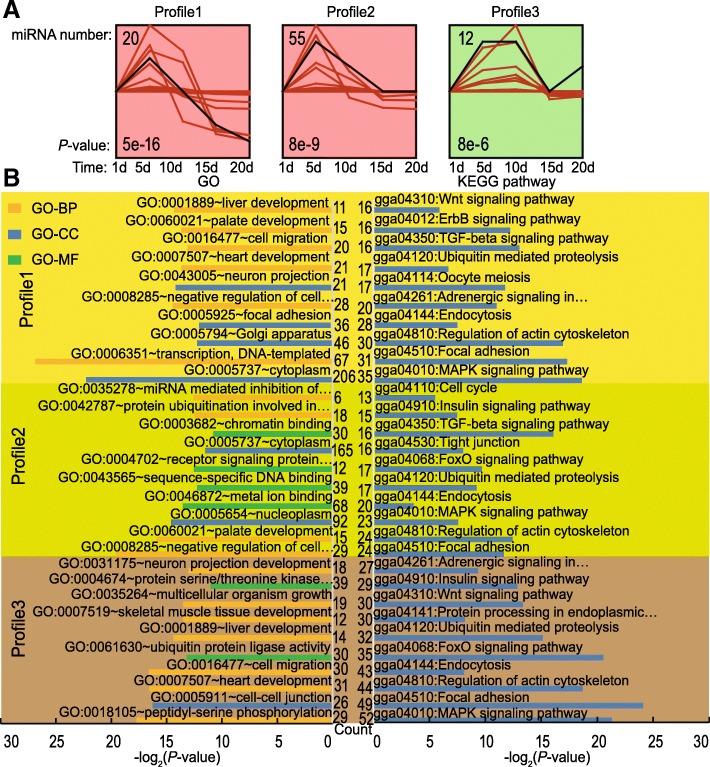
The miRNA expression analysis by STEM clustering. **a** The profiles that had a statistically significant *P-*value and a number of miRNAs assigned using the ‘log normalize data’ option, with all other settings set to the defaults. Statistically significant profiles that were similar formed a cluster of profiles, and were shown in different color(s). **b** Gene Ontology (GO) and KEGG pathway analysis of potentially targeted miRNAs

Exosomal miRNAs expression pattern across the lactation period in PM were assessed by the analysis of sequencing data using the Short Time-series Expression Miner software (STEM) algorithm. The STEM clustering tool assigned each miRNA to the model profile that most closely matched its temporal expression profile [36]. Three significant model profiles (profiles 1, 2 and 3) marked with colors from the 50 distinct expression patterns were generated (Fig. 5a and Additional file 7: Table S4). Profiles 1, 2 and 3 comprised 20, 55 and 12 miRNAs, respectively. Shown with the same color, profiles 1 and 2 were characterized to form a cluster of similar profiles. Interestingly, the expression level of all three profiles gradually increased from 1 to 5 d, peaking on day 5 and then altered. However, the expression level for profiles 1 and 2 subsequently decreased to 20 d and 15 d, respectively. While, the expression level of the profile 3 remained high till 10 d and then decreased to 15 d; then it increased again to 20 d. These results indicate that the expressed miRNAs within these profiles may play different roles for the development of squabs. Therefore, we carried out the prediction of potential target genes for these miRNAs. As depicted in Fig. 5b, the target genes of miRNAs in profile 1 and 2 are mainly involved in cell migration (GO: 0016477), liver development (GO: 0001889), skeletal muscle tissue development (GO: 0007519) and mitogen activated protein kinase (MAPK) signaling pathway (gga04010), Wnt signaling pathway (gga04310), insulin signaling pathway (gga04910). Other functional terms including focal adhesion (gga04510), regulation of actin cytoskeleton (gga04810), endocytosis (gga04144) were also identified. Meanwhile, the target genes of miRNAs in profile 3 were enriched in Golgi apparatus (GO: 0005794), neuron projection (GO: 0043005) and heart development (GO: 0007507). The above results further suggest that the exosomal miRNAs in PM carry out array of important function in development of squabs.

## Discussion

To date, no studies have investigated miRNA expression profiles in PM. Hence, to the best of our knowledge, this is the first report to identify exosomal miRNAs in PM. Because PM is regarded as indispensable for the growth and development of squabs, numerous studies have explored its nutritional composition [8–10]. Although the physiological synthesis and secretion of PM and mammalian milk differ, both provide similar nourishment for the growth and development of offspring.

Previous studies have demonstrated the existence of many exosomal miRNAs in mammalian milk [22–24]. Interestingly, we observed that PM also contains large amounts of exosomes, similar to those of mammalian milk, in the present study. Furthermore, a considerable proportion of miRNAs found in mammalian milk were also present in PM, which is consistent with previous reports on breast milk from humans [22, 37], pigs [23, 38], cows [39], and giant pandas [24].

Of these, miR-148a-3p, a potential biomarker for the quality control of mammalian milk, showed relatively high expression levels (in the top 10) in PM across five lactation stages. This indicated that it may be stably expressed and evolutionarily conserved from mammalian milk. Similarly, other miRNAs such as miR-21 and miR-26a were also enriched in PM; these are regarded as potential biomarkers for the quality control of raw milk and other milk-related products [39]. Furthermore, expression of the conserved miRNAs between PM and breast milk from humans and bovine was positively correlated (Pearson’s *r* = 0.60 and 0.62, respectively), while the average correlation between mammalian breast milk groups was 0.68. Based on these findings, it is tempting to speculate that pigeon ‘lactation’ and mammalian lactation generate similar functional products although they evolved independently [1].

The process of PM production begins when the germinal cell layer of the crop rapidly proliferates, followed by desquamation of lipid-rich cornified epithelium and subsequent accumulation in the crop lumen [6]. Our results suggest that some exosomal miRNAs in PM have a regulatory role in the proliferation and desquamation of crop cells. For instance, miR-21 in PM maintained high expression levels during lactation periods. Previous studies showed that miR-21 is one of several oncogenic miRNAs that control tumor cell proliferation, migration, and invasion [40, 41]. miR-21 was also found to be up-regulated by replicative and stress-induced senescence, despite being described as oncogenic [42], which could be associated with the hypoxic stress caused by a lack of blood supply to the rapidly proliferating germinal cell layer of the pigeon crop [7].

miR-205a is another miRNA that was abundantly expressed in PM. Previous reports revealed that it promoted the migration of keratinocytes via lipid phosphatase SHIP2, and that it was involved in regulation of the actin cytoskeleton and cell migration [43]. Of note, the accumulation of neutral lipids in keratinocytes is a unique trait of avian species [44], and pigeons were shown to utilize this ability to produce lipid-rich PM in their crop [7]. Furthermore, miR-21, miR-26a, and miR-27b, which were enriched in PM in this study, were previously shown to be associated with the adipogenic differentiation of adipose cells [45, 46]. Moreover, miR-22-3p, miR-148a-3p, and miR-26a-5p were highly expressed in chicken hepatocytes and exhibited critical roles in lipid metabolism [47]. Taken together, these results suggest that the highly expressed miRNAs in PM are likely to be associated with various important physiological functions, and may play essential roles in the generation of PM.

Through the analysis of miRNA expression patterns in PM, we identified three significant model profiles. GO and KEGG analyses of the putative target genes of highly expressed miRNAs in these three profiles revealed that enriched pathways were associated with organ development, particularly that of the heart, liver, and nervous system, and growth-related pathways including insulin, MAPK, and Wnt signaling pathways. These findings suggest that exosomal miRNAs in PM promote the growth and development of squabs.

Previous studies on the composition of PM indicated that it comprises proteins and lipids [11]. Hu et al. reported that the insulin signaling pathway IRS1/Akt/TOR regulates the synthesis of crop ‘milk’ protein [48], which is consistent with our KEGG analysis results. Moreover, the MAPK and Wnt signaling pathways may act on the stem cell population that gives rise to the proliferative germinal epithelium, which subsequently differentiates into keratinocytes that eventually form PM [7]. Additionally, the fatty acid precursors of crop triglycerides are likely obtained through the blood supply from oxidized triglycerides, which are transported from the liver or adipose tissue as fatty acids or very-low density lipoproteins that enter the cell by endocytosis [7]. Our KEGG pathway analysis further revealed that the endocytosis pathway is also enriched in exosomal miRNAs of PM.

In summary, these results indicate that PM miRNAs with different expression patterns play important regulatory roles in the synthesis and secretion of PM and the subsequent development of squabs.

## Conclusions

In the present study, we have comprehensively enlightened and elucidated the distribution and expression profiles of exosomal miRNAs in PM and identified conserved miRNAs across species by small RNA-seq analysis, expanding the scope of the resources available to PM. Notably, similar to mammalian milk, a significant proportion of immune and growth-related miRNAs were present and enriched in PM. Besides, we also identified certain highly expressed miRNAs that may be involved in biosynthesis of PM; it is tempting to infer that our findings, therefore, prelude further detailed investigations elucidating the underlying essential roles of these highly expressed miRNAs. Nevertheless, based on our results, it is fascinating to speculate that the exosomal miRNAs in PM may be another factor affecting the development and growth of squabs.

## Methods

### Pigeon ‘Milk’ (PM) collection

The healthy pigeon squabs (*Columba livia*) were obtained from a pigeon farm in Wenjiang, Chengdu, Sichuan, China. PM samples were collected from squabs at five time points (1, 5, 10, 15 and 20 days after birth). As soon as the squabs were fed with PM, they were immediately anaesthetized with ether to minimize distress and subsequently euthanized. In brief, the pigeon squabs were respectively transferred to inverted beakers with cotton soaked in ether for about 30s. Subsequently, the squabs were euthanized by cervical dislocation. After 75% alcohol disinfection, their crops were surgically slit open by an incision of 2 cm in length. PM samples were collected from the crop of squabs into 15 ml sterile tubes and stored at − 80 °C for further analyses.

### Preparation of exosomes

For the preparation of exosomes, the serial centrifugation and ultracentrifugation procedures were conducted as previously described [38], with minor modifications. Briefly, the PM was purified and stirred in phosphate buffered saline (PBS). PM samples were cleared of large aggregates as well as cell debris by sequential centrifugation at 2000×g for 30 min at 4 °C. The supernatant was sequentially subjected to centrifugations at 12,000×g for 45 min at 4 °C to remove other detritus. Filtration procedures were applied with syringe filters (0.22 μm) before the supernatant was prepared by ultracentrifugation at 160,000×g for 90 min. The final fraction was resuspended in 250 μl of PBS.

### Atomic force microscope (AFM)

To characterize the morphology of isolated exosomes, PMs were diluted 1: 100 in deionized water and absorbed to freshly cleaved mica sheets for 20 min. The surplus solution was removed through careful absorption with a filter paper, and the mica was further dried before detection. Surface morphology was examined under an atomic force microscope (Asylum Research MFP-3D-Bio, Digital Instruments Inc., Santa Barbara, CA) as described by Sharma et al. [49].

### Western blotting

Pigeon crop tissue and PM exosomes were lysed by protein lysis buffer (RIPA, Beyotime, Shanghai, China) supplemented with a protease inhibitor (Pierce, Rockford, IL, USA). The protein concentration was quantified by the BCA method. The lysates were boiled in SDS loading buffer (Beyotime), and were electrophoresed in 10% SDS-polyacrylamide gels and then transferred onto PVDF membranes (Millipore, USA). After blocking in 5% skim milk, the membranes were incubated with rabbit anti-alpha Tubulin (1: 1000, Abcam) or mouse anti-CD63 (1: 1000, Abcam) overnight at 4 °C. Then the PVDF membranes were washed with Tris-buffered saline with Tween 20 (TBST) and hybridized with Horseradish peroxidase-conjugated anti-rabbit or anti-mouse IgG, which was used as a secondary antibody (diluted 1: 5000 in TBST) for 2 h at RT. After washing three times, the protein bands were visualized with chemiluminescence reagents (Bio-Bad Laboratories) and scanned using ChemiDOC XRS instrument (Bio-Rad Laboratories).

### Small RNA library preparation and sequencing

Exosomes were isolated from PM by serial differential centrifugation as previously described and collected in 250 μl PBS. Total RNA was extracted using Trizol-LS (Invitrogen, CA, USA) as per manufacturer’s instructions. The quality of RNA was examined by 1% agarose gel electrophoresis and further determined by the Agilent 2100 Bioanalyzer with RNA 6000 Nano LabChip Kit (Agilent Technologies, Santa Clara, CA). For each PM library, small RNA ranging from 15 to 36 nt in length was purified by polyacrylamide gel electrophoresis and ligated using proprietary adaptors. The modified small RNA was then reverse-transcribed to cDNA and amplified by PCR. Finally, the libraries were sequenced on an Illumina HiSeq 2500 platform. We also downloaded 16 breast milk libraries of three mammals from NCBI’s Gene Expression Omnibus (GEO) Fdatabase, including human (GSE32253, *n* = 4) [22], bovine (GSE55144, *n* = 4) [50] and pig (GSE36590, *n* = 8) [23].

### Identification of pigeon miRNAs and STEM analysis

To identify the pigeon miRNAs, initial sequence was subjected to a series of stringent filters (such as removing low quality-reads, repeated sequences and adaptor sequences) and the output was called clean data. Filtered sequences were then mapped to pigeon reference genome (*Columba livia,* ColLiv2) (https://www.ncbi.nlm.nih.gov/assembly/GCA_001887795.1) with stringent criteria (0 mismatch in the first 18 bp) using Bowtie software [51]. Since no exact annotation of pigeon miRNAs was recorded in miRBase 21.0, we performed alignment between the extended genomic sequence of all mappable reads and mature miRNA sequences of chicken (*Gallus gallus)*, zebra finch (*Taeniopygia guttata*) and other mammals allowing no mismatch. Likewise, novel miRNAs were further predicted using the miRDeep2 core algorithm. For each sample, counts were first normalized by the total count of mappable reads which is denoted as reads per million (RPM), then standardized by the formula log_2_ (RPM + 1), which allowed unbiased comparison among samples. For each miRNA, the normalized number of counts was compared between groups.

A time-series analysis of the miRNA expression data was performed using STEM software (Short Time-series Expression Miner) http://www.cs.cmu.edu/~jernst/stem/ [52]. By STEM analysis, each miRNA was assigned to the model profile most closely matched to its time series based on the correlation coefficient. STEM was run using the log normalize data option, with all other settings set to the defaults.

### Identification of orthologous miRNAs

As miRNAs that share highly similar sequences are likely to have similar functions, orthology groups of multi-species miRNAs were mainly constructed based on their similarity over their entire length. The identification of orthologous procedures were conducted with reference literatures [53, 54]. At first, we performed an all-against-all Blast search of miRNA precursors detected in all samples consisting of PM and breast milk libraries derived from human, bovine and pig. Blast hits with > = 75% of both precursor aligned and an identity of > = 70% were retained. Secondly, the miRNAs paired through blast hits were then grouped into homologous families using hcluster_sg [55]. To further eliminate the duplication miRNA genes in each homologous family and get the 1–1-1 orthology, we brought in a genomic location-matching approach based on BED tools [56], in which only miRNAs with the maximal overlap of precursor genomic coordinates were kept for each orthologous family.

### Prediction and functional annotation of miRNA target genes

The algorithm TargetScan (http://www.targetscan.org/vert_71/) was applied to annotate miRNA target genes [57]. Gene Ontology (GO) and KEGG pathway analyses of target genes were performed using DAVID bioinformatics resources (https://david.ncifcrf.gov/) [58], with the Chicken *(Gallus gallus)* genome was selected as the background reference, which is the most closely related species for pigeon of the currently available entries in DAVID database. DAVID gene ontology enrichment was regarded as significant if the Benjamini *P-*value was smaller than 0.05.

### Q-PCR

The expression changes of randomly selected miRNAs were determined by q-PCR approach. The cDNA was synthesized from miRNAs using the Mir-X™ miRNA First Strand Synthesis Kit (Clontech, Dalian, China) according to the supplier’s protocol. Quantitative PCR was carried out using SYBR Premix Ex Taq II (Takara, Dalian, China) on the CFX96™ Real-Time PCR Detection System (Bio-Rad, CA, USA). U6 snRNA were simultaneously used as internal control genes. The miRNA forward primers were obtained commercially from BGI (Shenzhen, China) and the universal reverse primer for miRNAs was offered by the Mir-X™ miRNA First Strand Synthesis Kit (Clontech, Dalian, China). The primer sequences are provided in Additional file 8: Table S5. Each miRNA sample was analyzed in triplicate and the 2^−ΔΔCt^ method was used to calculate the relative expression levels of objective miRNAs.

### Statistical analysis

All data from q-PCR are presented as means ± SD. Pearson’s correlation was used to determine the relationship between the small RNA-seq and q-PCR approaches. The *χ*^2^-test was used to determine statistical significance. A value of *P* < 0.05 was considered as significant (* *P* < 0.05, ** *P* < 0.01).

## Additional files

Additional file 1: **Figure S1.** Agilent 2100 analysis of total RNA from exosomes in PM. (PDF 952 kb)

Additional file 2: **Table S1.** The sequencing results. (XLSX 9 kb)

Additional file 3: **Figure S2.** Length distribution and expression distribution of identified exosomal miRNAs in PM. (PDF 396 kb)

Additional file 4: **Table S2.** The mature miRNA group identified in PM and other three mammals. (XLSX 645 kb)

Additional file 5: **Figure S3.** Q-PCR validation of miRNAs. (PDF 388 kb)

Additional file 6: **Table S3.** The conserved miRNA group identified in 4 species. (XLSX 155 kb)

Additional file 7: **Table S4.** miRNAs belonging to three Profiles as assigned by the STEM clustering tool. (XLSX 20 kb)

Additional file 8: **Table S5.** The Primer sequences of q-PCR experiments. (XLSX 9 kb)

## Abbreviations

AFM: Atomic force microscope
GALT: Gut associated lymphoid tissue
GO: Gene ontology
hASCs: Human adipose tissue-derived mesenchymal stem cells
KEGG: Kyoto encyclopedia of genes and genomes
PBS: Phosphate buffered saline
PM: Pigeon ‘Milk’
RPM: Reads per million
STEM: Short Time-series Expression Miner software
TBST: Tris-buffered saline with tween 20
