# Supplementary figures and images for: Exploration of exosomal microRNA expression profiles in pigeon ‘Milk’ during the lactation period
Source: BMC Genomics. 2018 Nov 20;19:828. doi: 10.1186/s12864-018-5201-0 (PMC6245878; doi:10.1186/s12864-018-5201-0)

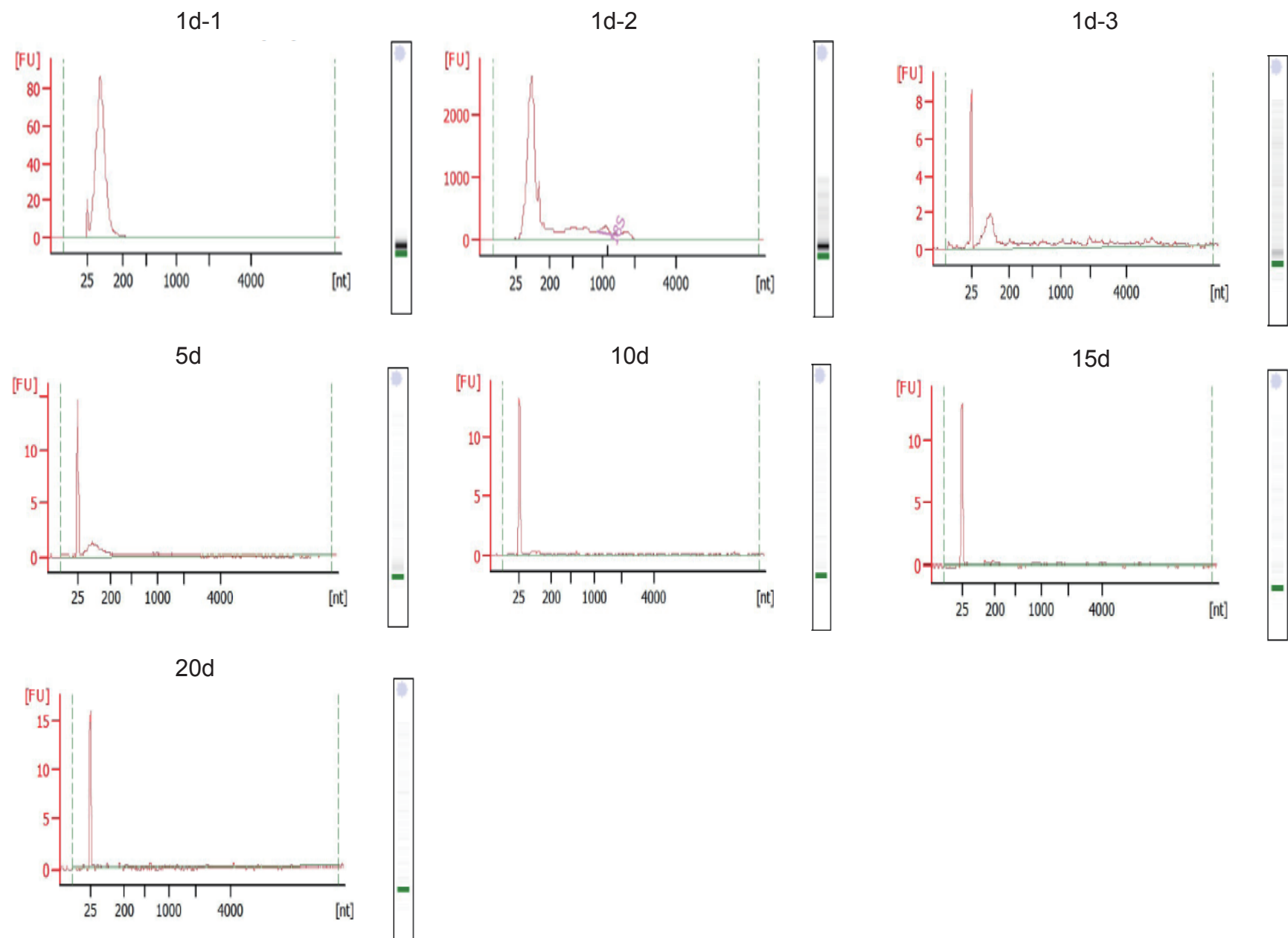

Figure S1. Agilent 2100 analysis of total RNA from exosomes in PM

Supplement: Supplementary file 1 — Figure S1. Agilent 2100 analysis of total RNA from exosomes in PM. (PDF 952 kb) [file 12864_2018_5201_MOESM1_ESM.pdf]

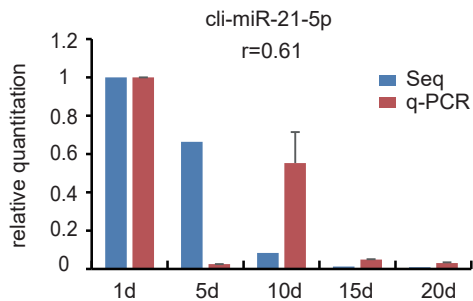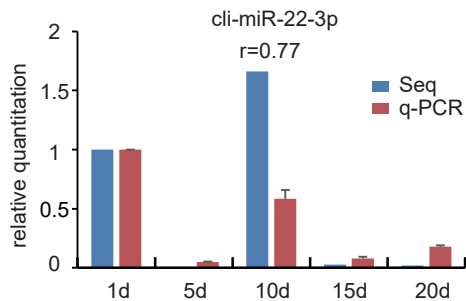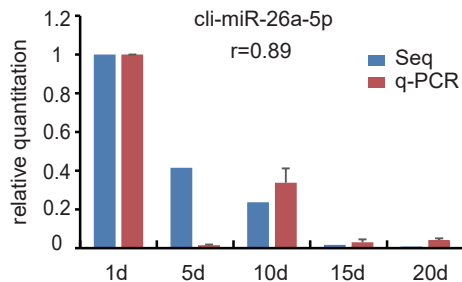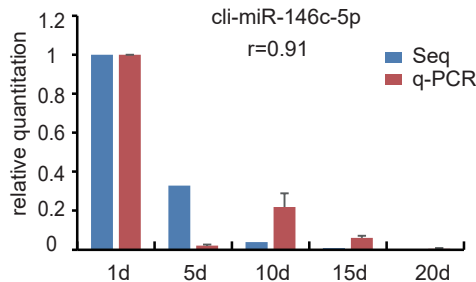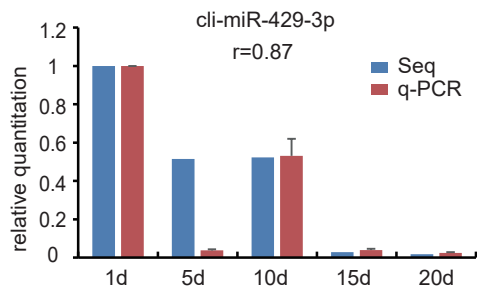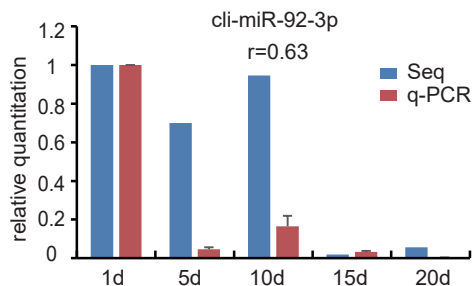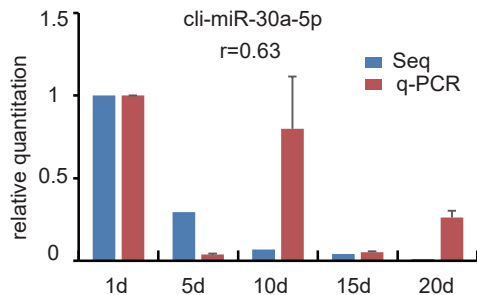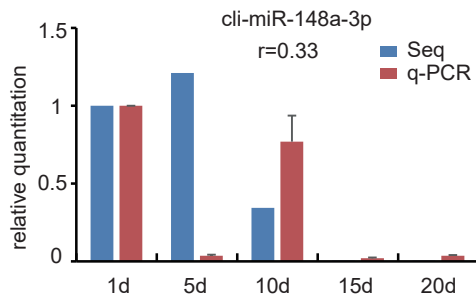

Figure S3. Q-PCR validation of miRNAs.

Supplement: Supplementary file 5 — Figure S3. Q-PCR validation of miRNAs. (PDF 388 kb) [file 12864_2018_5201_MOESM5_ESM.pdf]
