# Supplementary material for: Exploration of exosomal microRNA expression profiles in pigeon ‘Milk’ during the lactation period
Source: BMC Genomics. 2018 Nov 20;19:828. doi: 10.1186/s12864-018-5201-0 (PMC6245878; doi:10.1186/s12864-018-5201-0)
Supplement: Supplementary file 3 — Figure S2. Length distribution and expression distribution of identified exosomal miRNAs in PM. (PDF 396 kb) [file 12864_2018_5201_MOESM3_ESM.pdf]

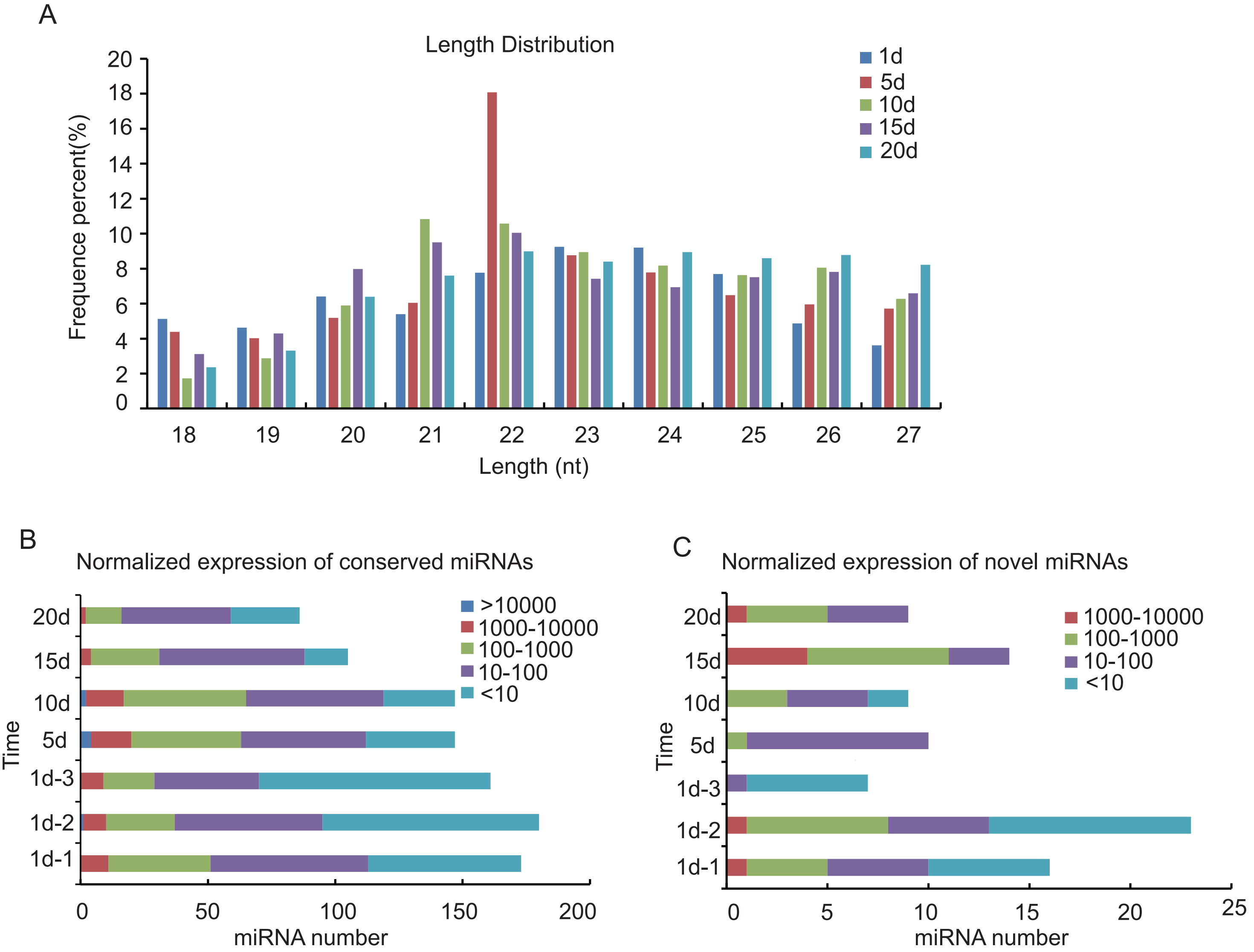

Figure S2. Length distribution and expression distribution of identified exosomal miRNAs in PM.
